# F-actin-dependent activation of NADPH oxidase 5 by the vacuolar-type ATPase

**DOI:** 10.1016/j.jbc.2026.113546

**Published:** 2026-09-11

**Authors:** Gwen J. Heywood, Samantha M. Richter, Archi S. Paul, Elizabeth A. Sweeny

**Affiliations:** Department of Biochemistry, The Medical College of Wisconsin, Milwaukee, Wisconsin, USA

**Keywords:** NOX5, v-ATPase, actin, superoxide ion, cytoskeleton, reactive oxygen species, PSN-1, cell migration

## Abstract

NADPH oxidase 5 (NOX5) is a transmembrane oxidative signaling enzyme that produces superoxide in response to increases in intracellular calcium. NOX5 has been shown to play essential roles in processes such as specialized cell differentiation, proliferation, and cell contraction, while its overexpression and hyperactivation are associated with human diseases, including cardiovascular disorders, cancer, and diabetic kidney disease. Despite its importance in human health and disease, the regulatory factors and mechanistic signaling details of NOX5 remain incompletely resolved. We previously reported that chemical stabilization of F-actin by jasplakinolide induces a superoxide burst by NOX5 independent of changes in intracellular calcium. Here, we show that this F-actin-dependent activation of NOX5 superoxide production relies on the vacuolar-type ATPase (v-ATPase). In cells, jasplakinolide treatment stabilizes a complex comprising actin, actin-binding proteins, the v-ATPase, and NOX5. Chemical inhibition of the v-ATPase or v-ATPase subunit knockdown decreases NOX5 superoxide production in response to jasplakinolide while only minimally affecting NOX5 superoxide production in response to increases in intracellular calcium or NOX5 phosphorylation. Finally, in untreated cells, the v-ATPase and NOX5 localize to the cell leading edge and contribute to cell migration. Our findings represent a novel mechanism of NOX5-mediated superoxide production by the v-ATPase and provide a new foundation for developing strategies to disrupt protein interactions and activities that drive aberrant cell migration.

Elevated levels of reactive oxygen species (ROS) cause oxidative stress, damage cells and tissues, and contribute to disease initiation and progression. However, ROS also play a critical role as signaling molecules, and are required for several physiological processes. NADPH oxidases (NOX) are a primary source of ROS in the cell and serve dual roles in physiological signaling and pathological damage. Although largely structurally conserved, their tissue expression, intracellular localization, and modes of regulation are highly divergent (1). In fact, even when expressed in the same cell, they have been shown to play distinct functional roles (2, 3).

NOX5 is the most recently discovered member of the NOX family. While other family members rely on maturation factors and/or protein partners to form an active protein complex, NOX5 produces a superoxide burst in response to calcium (Ca^2+^) binding to its EF-hand domains (4, 5) without the need for protein partner interactions. NOX5 is found in most mammals, including humans, but is absent from mice and rats. This has delayed our understanding of its diverse physiological functions; however, recent discoveries have revealed that NOX5 plays a critical role in vascular contraction (6), sperm motility, and viability (7), and is required for monocyte differentiation into dendritic cells (8), and differentiation and maturation of oligodendrocytes (3). Moreover, recent studies suggest that increased expression and hyperactivation of NOX5 play a role in the development of cancer (9, 10), diabetes (11, 12), hypertension (13, 14), and cardiovascular disorders (15, 16).

Superoxide production by NOX5 is regulated through a combination of effectors: inhibitors, activators, and enhancers. Proteins known to negatively regulate NOX5 include caveolin-1 (17), Hsp90 (18), and inactive NOX5 isoforms (19). Inhibitory posttranslational modifications include S-nitrosation (20) and oxidative modification (21). NOX5 activation can be stimulated by increases in intracellular Ca^2+^ (4, 5), and by phosphorylation by kinases including protein kinase C α (PKCα) (22, 23, 24), calcium and calmodulin-dependent protein kinase II (CAMKII) (25), and c-Abl kinase (26). Activation by phosphorylation is proposed to occur through increasing the Ca^2+^ sensitivity of NOX5, allowing for activation at resting Ca^2+^ levels. Enhancers are factors that increase NOX5 superoxide production upon activation. These include the peptide hormones endothelin-1 and angiotensin II (27), intracellular heme levels (18), and nitric oxide (through the formation of heme-NO) (28).

We recently discovered that NOX5 and actin interact in the cell (29), and that the addition of jasplakinolide, a naturally occurring cyclic peptide found in *Jaspis johnstoni* that stabilizes F-actin (30), stimulates NOX5 superoxide production independent of increases in Ca^2+^ (29). We hypothesized that this F-actin-dependent activation may be occurring physiologically in regions of the cell undergoing large, transient increases in F-actin, such as at the cell leading edge. This hypothesis led us to investigate a role for NOX5 in the migration of the pancreatic cancer cell line PSN-1, in which NOX5 is overexpressed, and indeed, we found that siRNA-mediated knockdown of NOX5 in these cells decreased cell migration (29). Despite these findings, the mechanistic basis for the F-actin-dependent activation of NOX5 superoxide production, and a direct connection to the events at the leading edge of migrating cells, remained unresolved.

To address these gaps, we first assessed the contribution of NOX5 phosphorylation to its F-actin dependent activation. We found no evidence that the NOX5 superoxide production induced by jasplakinolide was associated with either phosphorylation or changes in Ca^2+^ (29), indicating that it was initiating NOX5 superoxide production through an unknown, novel mechanism. Instead, we identified a protein complex stabilized by jasplakinolide that included actin, actin-binding proteins, NOX5, and subunits of the vacuolar-type ATPase (v-ATPase). The v-ATPases are ATP-dependent proton pumps that can localize to a variety of cellular membranes and play essential roles in several physiological processes, including lysosomal acidification (31). The v-ATPase is a large complex comprised of two hetero-oligomer domains: a membrane-embedded proton channel, V_0_, and a cytosolic ATPase, V_1_ (32, 33). Activity is controlled by regulated assembly and localization (31, 32, 33, 34, 35, 36, 37, 38), with ATP hydrolysis by V_1_ driving proton translocation across the membrane through V_0_. While this is the first study to show a connection between the v-ATPase and NOX5 activity, there is precedent for a proton pump regulating NOX activity. The hydrogen voltage-gated channel 1 (Hν1) is essential for phagocytic NOX ROS production (39, 40) and has been shown to be coupled to NOX5 activity in human spermatozoa (41). Therefore, we assessed the effect of chemical inhibition of the v-ATPase and siRNA-mediated knockdown of v-ATPase subunits and found that both specifically decreased the F-actin dependent activation of NOX5 superoxide production by jasplakinolide. Furthermore, in untreated cells, both NOX5 and subunits of the v-ATPase partially localized to the cell leading edge, and both were found to contribute to PSN-1 cell migration. Together, our findings suggest that an F-actin dependent complex at the cell leading edge recruits NOX5 and the v-ATPase, which then collaborate to drive directional cell migration.

## Results

### F-actin-dependent activation of superoxide production by NOX5

Previous work has shown that superoxide production by NOX5 can be activated through increases in intracellular calcium (4, 5) and NOX5 phosphorylation by kinases including PKCα (22, 23, 24), c-Abl (26), and CAMKII (25). This activation can be initiated experimentally by the addition of ionomycin Ca^2+^, or phorbol 12-myristate 13-acetate (PMA), respectively. Our previous study identified an additional mode of NOX5 activation, through an unknown mechanism, induced by the addition of the F-actin stabilizing compound jasplakinolide (29). To examine how activation of NOX5 superoxide production by jasplakinolide compared to that initiated by ionomycin Ca^2+^ or PMA, we measured superoxide production in human embryonic kidney 293 (HEK293) cells that stably express NOX5β using the extracellular superoxide sensor L-012 (18, 20, 42, 43) with all three activating compounds. The kinetics of superoxide production differ between activation modes: stimulation with ionomycin Ca^2+^ results in a superoxide burst initiated within seconds and resolved over minutes, whereas activation with PMA initiates superoxide production within minutes and can be sustained for over an hour. Therefore, we measured superoxide production after automated addition of 1 μM ionomycin Ca^2+^ or 1 μM jasplakinolide for 300 s (Fig. 1*A*), or after manual addition of 100 nM PMA or 1 μM jasplakinolide for 40 min (Fig. 1*B*). Jasplakinolide addition stimulated a bimodal pattern of superoxide production by NOX5, with a small initial burst, likely due to the effect of the compound on Ca^2+^ release from the ER (29), followed by a secondary phase of superoxide production that occurs over minutes (Fig. 1, *A* and *B*). No superoxide was detected under any experimental conditions in HEK293 cells that do not express NOX5 (Fig. 1, *A* and *B*), and transient transfection of HEK293 cells with NOX5β displayed similar kinetics (Fig. S1, *A*–*C*). F-actin can act as a signaling hub, localizing posttranslational modification machinery, including kinases (44, 45), and we have previously reported an interaction between NOX5 and actin (29). In combination with the observed similarity in the kinetics of NOX5 superoxide production in response to jasplakinolide and PMA (Fig. 1*B*), we hypothesized that F-actin stabilization by jasplakinolide was facilitating NOX5-kinase interactions, resulting in NOX5 phosphorylation and subsequent activation. To explore this possibility, we used siRNA-mediated knockdown of kinases known to phosphorylate NOX5 and assessed their effects on NOX5 superoxide production in response to 1 μM ionomycin Ca^2+^, 1 μM jasplakinolide, and 100 nM PMA (Fig. 1, *C*, *D*, *F* and *G*). Knockdown of PKCα (Fig. 1*E*) decreased PMA-stimulated superoxide production by NOX5 as expected (Fig. 1*D*), but had no effect on ionomycin Ca^2+^, or jasplakinolide-stimulated superoxide production (Fig. 1, *C* and *D*). Knockdown of c-Abl (Fig. 1*H*) had no effect on NOX5 superoxide production stimulated by any of the three compounds (Fig. 1, *F* and *G*), and we could not detect CAMKII protein in our HEK293 cells (Fig. S1*D*). To assess the possibility that an unknown kinase was responsible, we performed phosphoproteomics. HEK293 cells stably expressing NOX5β were treated with 1 μM jasplakinolide, 100 nM PMA, or dimethyl sulfoxide (DMSO) (vehicle control, x2) for 10 min. After cell lysis, phosphatase was added to one of the DMSO control conditions to dephosphorylate the sample, for four total conditions: 1) phosphatase-treated, 2) DMSO, 3) jasplakinolide, and 4) PMA. The samples were then subjected to immunoprecipitation using a NOX5 antibody and analyzed by mass spectrometry (MS) to identify NOX5 phosphorylation sites. Of the four samples, phosphorylated NOX5 peptides were only found in the PMA-treated sample at previously identified phosphorylation sites, S501 and S505 (24) (Fig. 1*I*). Together, these results suggested that the jasplakinolide-induced NOX5 superoxide production was not driven by NOX5 phosphorylation.

**Figure 1:**
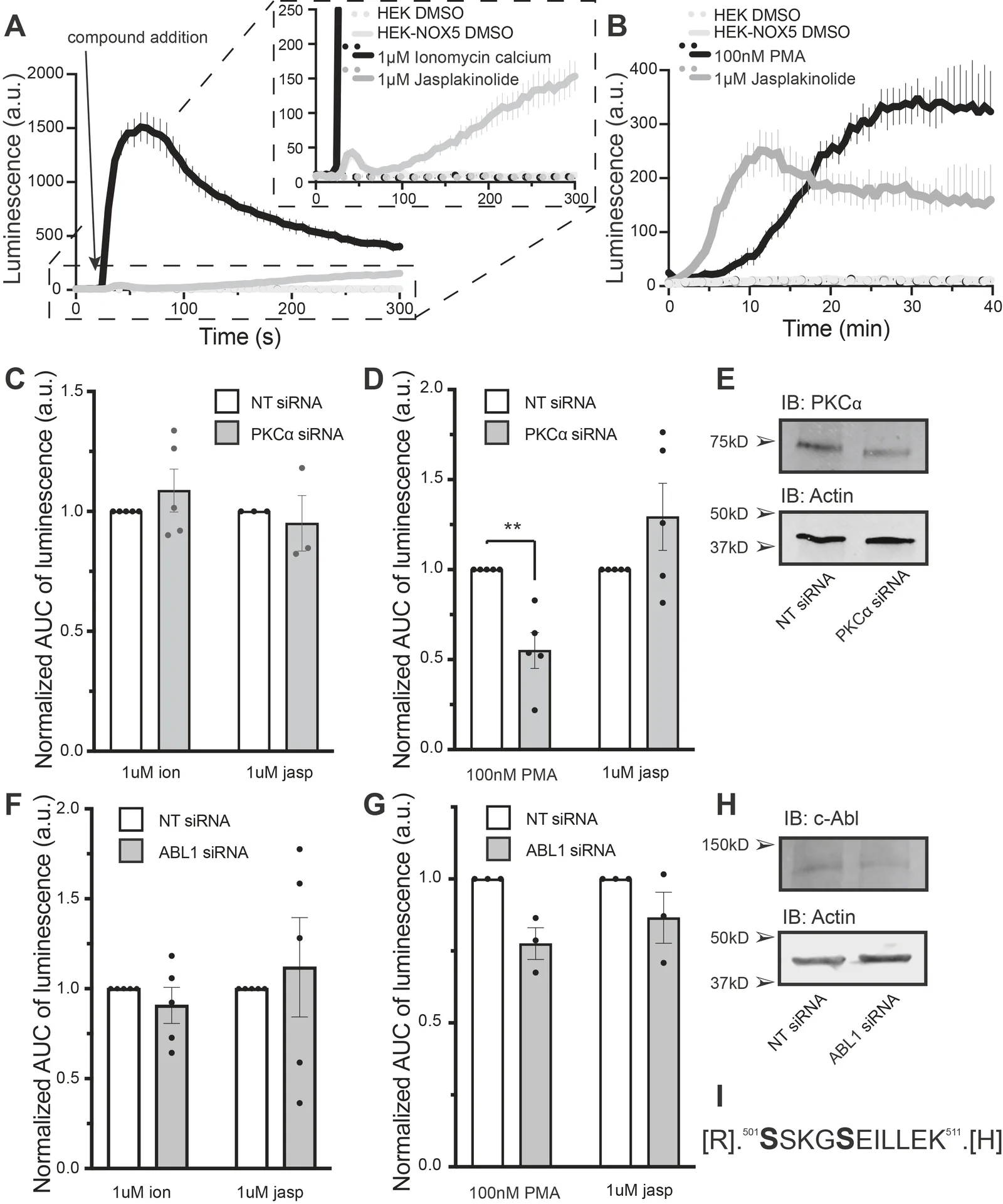
**Activation of NOX5 superoxide production by calcium, phosphorylation, and F-actin stabilization.** Superoxide levels were measured using the luminol analog L-012, which luminesces in the presence of superoxide. *A*, superoxide production by HEK293 cells and HEK293 cells stably expressing NOXβ (HEK-NOX5) after automated addition of DMSO (vehicle control), 1 μM ionomycin Ca^2+^, or 1 μM jasplakinolide measured for 300 s. *B*, superoxide production by HEK293 cells and HEK-NOX5 cells after manual addition of DMSO, 100 nM PMA, or 1 μM jasplakinolide measured for 40 min. *C* and *D*, quantification of NOX5 superoxide production (area under the curve) in HEK-NOX5 cells after transfection with nontargeting (NT) or PKCα-targeting siRNA after (*C*) automated addition of 1 μM ionomycin Ca^2+^ or 1 μM jasplakinolide (300 s) or (*D*) manual addition of 100 nM PMA or 1 μM jasplakinolide (40 min). Values represent means ± SEM, n = 3–5. Differences in NOX5 activity with NT *versus* PKCα-targeting siRNA were assessed using a two-tailed Student’s *t* test with ∗∗ denoting *p* < 0.001. *E*, representative Western blot of PKCα and β-actin expression with NT and PKCα-targeting siRNA transfection in HEK-NOX5 cells. *F* and *G*, quantification of NOX5 superoxide production in HEK-NOX5 cells after transfection with nontargeting (NT) or ABL1-targeting siRNA after (*F*) automated addition of 1 μM ionomycin Ca^2+^ or 1 μM jasplakinolide (300 s) or (*G*) manual addition of 100 nM PMA or 1 μM jasplakinolide (40 min). Values represent means ± SEM, n = 3 to 5. Differences in superoxide production with NT *versus* ABL1-targeting siRNA were assessed using a two-tailed Student’s *t* test (no significance). *H*, representative Western blot of c-Abl and β-actin expression with NT and ABL1-targeting siRNA transfection in HEK-NOX5 cells. *I*, NOX5 peptide identified by phosphoproteomics after 100 nM PMA treatment with phosphorylation sites in *bold*. DMSO, dimethyl sulfoxide; HEK293, human embryonic kidney 293; NOX, NADPH oxidase; PKCα, protein kinase C α; PMA, phorbol 12-myristate 13-acetate.

### Jasplakinolide-induced complex formation

Protein–protein interactions have been shown to play an important role in regulating NOX5 activity (17, 18, 19). Since jasplakinolide stabilizes F-actin, we hypothesized that it may stabilize an F-actin-dependent protein complex that results in NOX5 activation. To examine whether jasplakinolide treatment altered F-actin-dependent NOX5 protein interactions, we treated HEK293 cells stably expressing NOX5β with 1 μM jasplakinolide or DMSO for 10 min, then fractionated the cell lysates by glycerol gradient centrifugation. Odd fractions were subjected to SDS-PAGE and Western blot analysis (Fig. 2*A*). In the DMSO control, actin was found exclusively in the lower fractions, indicative of low molecular weight species (Fig. 2*A*). In contrast, jasplakinolide treatment resulted in actin bands found in fractions 21 and 23, indicating they were associated with a high molecular weight complex (Fig. 2*A*). NOX5 was also found in these fractions and its level appeared to be increased in fraction 23 (Fig. 2*A*). We performed MS analysis on fraction 22 to determine which proteins were enriched in the jasplakinolide treated sample compared to DMSO, analyzed the results using the STRING database for protein-protein association networks (46), and visualized them using Cytoscape (47) (Fig. 2*B*). A total of 47 proteins were identified as enriched in the jasplakinolidetreated sample compared to DMSO (Fig. 2*B*, Table S1). As expected, a large cluster (21 proteins) represented actin isoforms and actin-binding proteins, including cortactin (upper right, Fig. 2*B*). Another cluster (17 proteins) comprised mitochondrial translation machinery (lower left, Fig. 2*B*). A third cluster (four proteins) contained subunits of the cytosolic V_1_ domain of the v-ATPases. Interestingly, a recent study reported that partial loss of the v-ATPase mitigated the deleterious effects of mitochondrial translation defects (48). However, we were interested in a potential role for the v-ATPase in stimulating NOX5 superoxide production at the plasma membrane (PM) in response to F-actin stabilization. The v-ATPase is known to localize to the PM in specialized cell types and cancer cells (31) and has been shown to interact with actin and affect actin dynamics (49, 50, 51). Furthermore, the Hν1 proton pump has previously been shown to enhance activation of both NOX2 (39, 40) and NOX5 (41). Subunits of the V_0_ membrane domain were present at equal abundance in the DMSO- and jasplakinolide-treated samples (Table S1), suggesting that jasplakinolide treatment was increasing the association of V_1_ with V_0_ and assembly of functional v-ATPase. Coimmunoprecipitation studies found that treatment of HEK293 cells stably expressing NOX5β with 1 μM jasplakinolide for 2 min increased the association of the V_1_ subunit A1 (V1A1) with NOX5, actin, and a subunit of the V_0_ complex, D1, compared to the DMSO control (Fig. 2, *C* and *D*). Jasplakinolide treatment also increased the amount of cortactin coimmunoprecipitated with NOX5 (Fig. 2*E*), and the amount of actin coimmunoprecipitated with the V_1_ subunit C1 (V1C1), which is known to interact with actin (51) (Fig. 2*F*). We did not see any coimmunoprecipitation with cortactin and subunits of the v-ATPase (data not shown). Finally, buffer-based PM isolation revealed an increase in NOX5 at the PM after cells were treated with 1 μM jasplakinolide for 2 min (Fig. 2*G*), suggesting that this complex may be localized to the PM.

**Figure 2:**
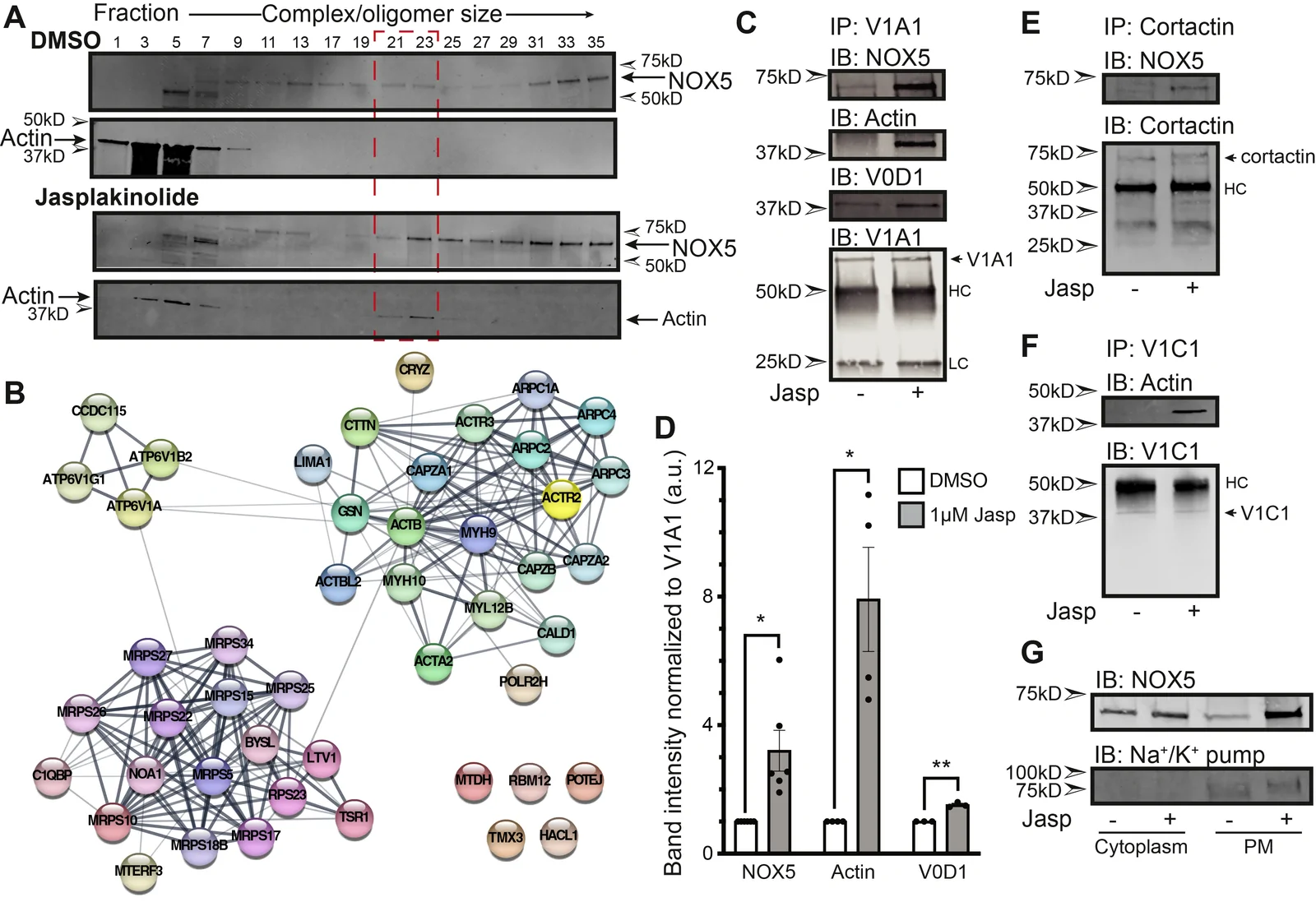
**Jasplakinolide treatment induces formation of a protein complex including actin, NOX5, and thev-ATPase.***A*, Western blot of odd-numbered glycerol gradient fractions of HEK-NOX5 cell lysates after treatment with DMSO (vehicle control) or 1 μM jasplakinolide for 10 min, probed for NOX5 and β-actin. Higher fraction numbers indicate higher molecular weight. Fractions that contain β-actin bands only in the jasplakinolide-treated sample are highlighted by a *dashed red box* (fractions 21&23). *B*, visualization of the proteins found to be enriched in fraction 22 in the jasplakinolide *versus* DMSO-treated HEK-NOX5 lysates. Proteins were identified by mass spectrometry, and enriched proteins analyzed using the STRING database for protein-protein association networks (46), and visualized using Cytoscape (47). *C*, representative Western blot, and *D*, quantification of coimmunoprecipitation of NOX5, β-actin, and the V_0_ subunit V0D1 with the V_1_ subunit V1A1 after treatment of HEK-NOX5 cells with DMSO or 1 μM jasplakinolide for 2 min. Values represent means ± SEM, n = 3–6. Differences in coimmunoprecipitated protein normalized to V1A1 levels were assessed using a two-tailed Student’s *t* test with ∗ denoting *p* < 0.05 and ∗∗*p* < 0.001. *E* and *F*, representative Western blots of coimmunoprecipitated NOX5 with cortactin (*E*) and actin with the V_1_ subunit V1C1 (*F*) after treatment of HEK-NOX5 cells with DMSO or 1 μM jasplakinolide for 2 min. *G*, representative Western blot of NOX5 and the sodium/potassium (Na^+^/K^+^) pump after treatment with DMSO or 1 μM jasplakinolide for 2 min and buffer-based separation of the plasma membrane. DMSO, dimethyl sulfoxide; HEK, human embryonic kidney; NOX, NADPH oxidase; v-ATPase, vacuolar-type ATPase; V0D1, V_0_ subunit D1; V1A1, V1 subunit A1.

### NOX5 and v-ATPase at the cell leading edge

NOX5 and the v-ATPase have previously been shown to partially localize to the PM, and to contribute to cell migration (29, 31, 52, 53). To determine whether these proteins localized to the cell leading edge in our system, we performed immunofluorescent confocal imaging studies in HEK293 cells stably expressing NOX5β, and compared the localization of NOX5 and V1A1 with cortactin, an actin-binding protein known to localize to the cell leading edge, which came up in our MS results for proteins enriched in fraction 22 (Fig. 2*B*), and showed an increased interaction with NOX5 upon jasplakinolide treatment (Fig. 2*E*). We hypothesized that while the complex containing NOX5, actin, actin-binding proteins, and the v-ATPase was stabilized by the addition of jasplakinolide, that it occurs physiologically in regions of the cell undergoing transient increases in F-actin, such as the leading edge of migrating cells. Therefore, these studies were performed with untreated cells. As expected, we observed cortactin at regions of cell membrane protrusions (Fig. 3) and found a strong overlap with NOX5 localization (Fig. 3*A*). Although V1A1 did not appear to directly overlap with cortactin, it was found in adjacent regions of the PM (Fig. 3*B*). These findings were in agreement with the coimmunoprecipitation data, which indicated a direct interaction of NOX5 and cortactin, but no interaction between V1A1 and cortactin.

**Figure 3:**
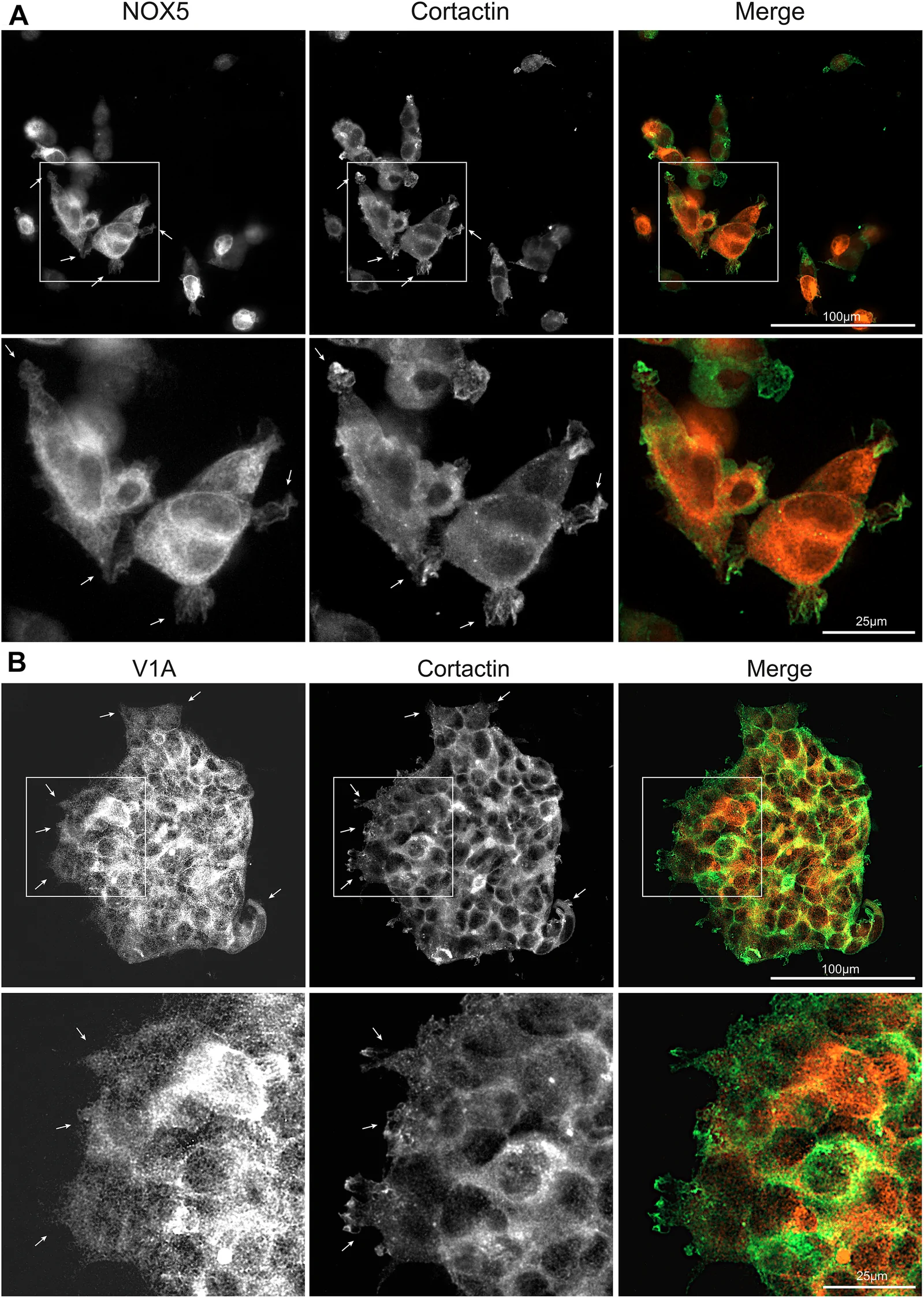
**NOX5, the V_1_ subunit V1A1, and cortactin at the leading edge of HEK-NOX5 cells.** Confocal immunofluorescent microscopy of (*A*) NOX5 and cortactin and (*B*) V1A1 and cortactin in HEK-NOX5 cells. *Grayscale images* are of individual channels (anti-NOX5 and anti-cortactin in *A*, anti-V1A1 and anti-cortactin in *B*). In merged images, cortactin is depicted in *green*, NOX5/V1A1 in *red*. The scale bar in top images represent 100 μm, the area highlighted in *white* is shown in the *lower panels* with a scale bar of 25 μm. *White arrows* indicate regions of the cell edge where there is staining of the proteins of interest. HEK, human embryonic kidney; NOX, NADPH oxidase; V1A1, V1 subunit A1.

### The role of the v-ATPase in F-actin-dependent activation of NOX5 superoxide production

To assess whether the interaction between the v-ATPase and NOX5 was responsible for the observed stimulation of NOX5 superoxide production upon addition of the F-actin stabilizing compound jasplakinolide, we employed both chemical inhibition of the v-ATPase with bafilomycin A (54) and siRNA-mediated knockdown of the v-ATPase V1 subunit V1C1 (Fig. 4). HEK293 cells stably expressing NOX5β were incubated with 100 nM bafilomycin A for 6 h, after which superoxide production was measured after automated addition of 1 μM ionomycin Ca^2+^ or 1 μM jasplakinolide for 300 s (Fig. 4, *A*–*C*), or after manual addition of 100 nM PMA or 1 μM jasplakinolide for 40 min (Fig. 4, *D*–*F*). Bafilomycin A treatment decreased jasplakinolide-stimulated NOX5 superoxide production in both the 300 s and 40-min assays (Fig. 4, *B*, *C*, *E*, and *F*), while it had no effect on ionomycin Ca^2+^ stimulated superoxide production (Fig. 4, *A* and *C*), and a small, but not statistically significant effect on PMA-stimulated superoxide production (Fig. 4, *D* and *F*). Bafilomycin A treatment for up to 6 h did not affect NOX5 protein levels (Fig. 4*G*). Sustained inhibition of the v-ATPase (24 h bafilomycin A treatment) decreased superoxide production in response to jasplakinolide even further (Fig. S2, *B*, *C*, *E*, and *F*); however, it also caused a slight decrease in superoxide production in response to both ionomycin Ca^2+^ and PMA (Fig. S2, *A*, *C*, *D*, and *F*) likely due to a decrease in NOX5 protein levels (Fig. S2*G*). Intriguingly, when superoxide production in response to PMA addition was measured for 120 min, it appeared that rather than decreasing superoxide production overall, as observed with ionomycin Ca^2+^ and jasplakinolide (Fig. S2, *A*, *B*, and *E*), pretreatment with bafilomycin A shifted the kinetics of PMA-induced superoxide production, resulting in a decreased initial rate, but sustained production (Fig. S2*D*).

**Figure 4:**
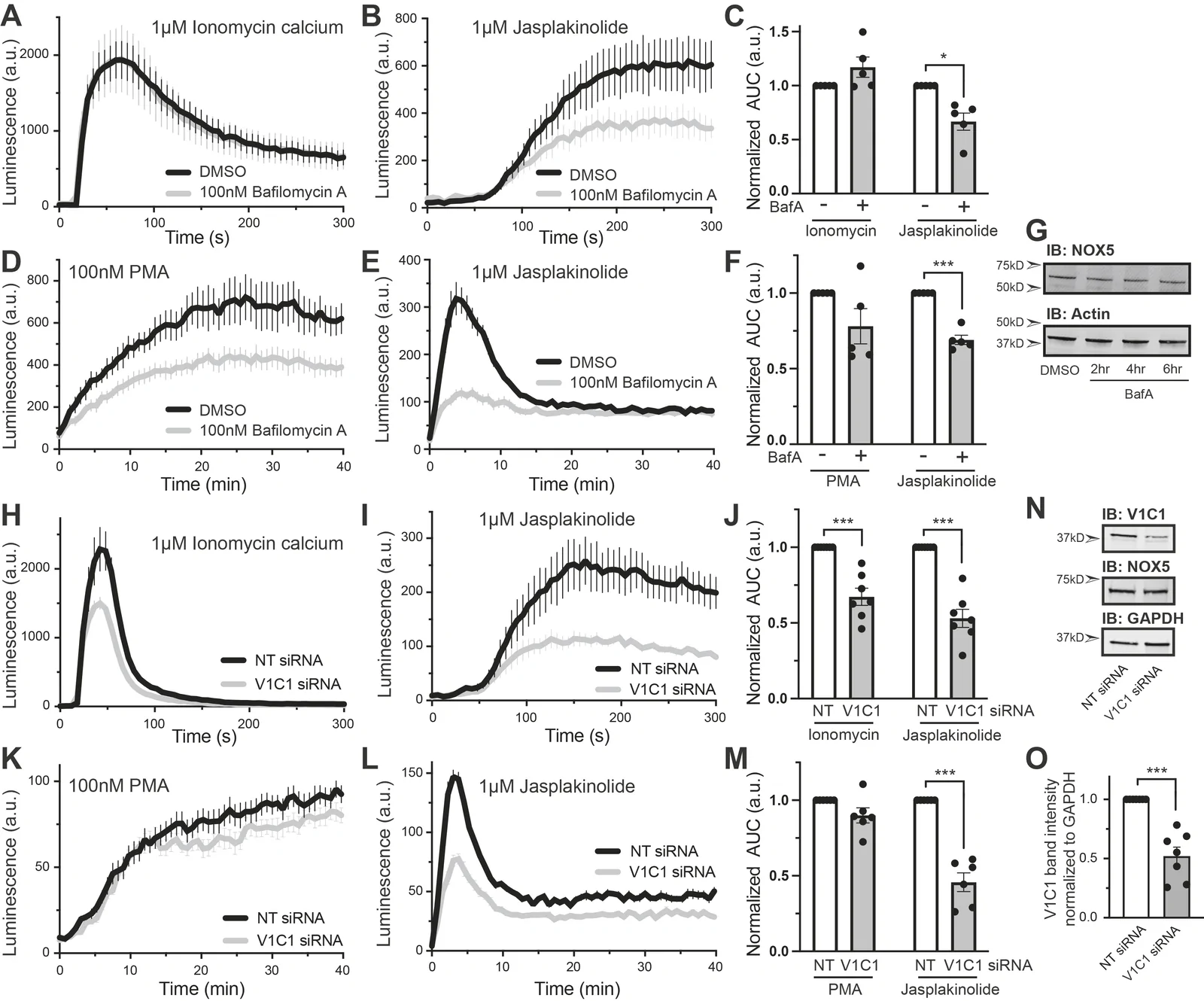
**Effect ofv-ATPase inhibition and subunit knockdown on NOX5 superoxide production.** Timecourse of superoxide production by HEK-NOX5 cells with automated addition of (*A*) 1 μM ionomycin calcium or (*B*) 1 μM jasplakinolide (300 s), or manual addition of (*D*) 100 nM PMA or (*E*) 1 μM jasplakinolide (40 min), after pretreatment with DMSO (vehicle control) or 100 nM bafilomycin A for 6 h, measured by L-012 luminescence. *C* and *F*, quantification of NOX5 superoxide production (area under the curve). Values represent means ± SEM, n = 5. Differences in superoxide after bafilomycin A treatment compared to DMSO were assessed using a two-tailed Student’s *t* test with ∗ denoting *p* < 0.05, and ∗∗∗*p* < 0.0001. *G*, representative Western blot probed for NOX5 and β-actin levels after treatment with DMSO or bafilomycin A for 2, 4, or 6 h. Timecourse of superoxide production by HEK-NOX5 cells with automated addition of (*H*) 1 μM ionomycin calcium or (*I*) 1 μM jasplakinolide (300 s), or manual addition of (*K*) 100 nM PMA or (*L*) 1 μM jasplakinolide (40 min), after transfection with nontargeting (NT) or V_1_ subunit V1C1-targeting siRNA measured by L-012 luminescence. *J* and *M*, quantification of NOX5 superoxide production (area under the curve). Values represent means ± SEM, n = 6–7. Differences in superoxide with NT *versus* V1C1-targeting siRNA were assessed using a two-tailed Student’s *t* test with ∗∗∗ denoting *p* < 0.0001. *N*, representative Western blot probed for V1C1, NOX5 and GAPDH. *O*, quantification of V1C1 protein levels with NT *versus* V1C1-targeting siRNA, values represent means ± SEM, n = 7, differences assessed using a two-tailed Student’s *t* test with ∗∗∗ denoting *p* < 0.0001. DMSO, diemthyl sulfoxide; HEK, human embryonic kidney; NOX, NADPH oxidase; v-ATPase, vacuolar-type ATPase; V1C1, V1 subunit C1.

We next assessed the effect of siRNA-mediated knockdown of the v-ATPase V1 subunit V1C1 on NOX5 superoxide production (Fig. 4, *H*–*J*, *K* and *L*). V1C1 knockdown was approximately 50% effective (Fig. 4, *N* and *O*) and had no effect on NOX5 protein levels (Fig. 4*N*). As observed with v-ATPase inhibition by bafilomycin A, knockdown of the V1C1 subunit decreased NOX5 superoxide production induced by jasplakinolide (Fig. 4, *I*, *J*, *L*, and *M*). There was no effect on PMA-stimulated superoxide production (Fig. 4, *K* and *M*), but a slight decrease in superoxide production in response to ionomycin Ca^2+^ (Fig. 4, *H* and *J*), which was not due to changes in Ca^2+^ (Fig. S3). While we observed small effects on PMA-stimulated (bafilomycin A, Fig. 4, *D* and *F*) and ionomycin Ca^2+^-stimulated (V1C1 knockdown, Fig. 4, *H* and *J*) superoxide production, only jasplakinolide-stimulated superoxide production was significantly decreased with both bafilomycin A inhibition and V1C1 knockdown (Fig. 4), suggesting that the v-ATPase plays a specific role in stimulating NOX5 superoxide production in response to F-actin stabilization.

### Contributions of the v-ATPase and NOX5 to PSN-1 cell migration

NOX5 and the v-ATPase have both been shown to play a role in cell migration (29, 31, 52, 53). Our findings suggest that NOX5 and the v-ATPase interact with actin at the PM, and that assembly and activation of the v-ATPase stimulate NOX5 superoxide production in an F-actin-dependent manner. These findings led us to hypothesize that they may be collaborating at the cell leading edge to drive cell migration. To assess this possibility, we employed the pancreatic cancer cell line PSN-1, which endogenously expresses NOX5 (55), and in which we have previously shown that siRNA-mediated knockdown of NOX5 decreases cell migration (29). Confocal immunofluorescence imaging confirmed that the v-ATPase V1 subunit V1C1 was localized at the cell leading edge of migrating PSN-1 cells (Fig. 5*A*). To determine the role of the v-ATPase and NOX5 in PSN-1 cell migration, we employed a scratch assay in which images were taken every hour for 18 to 22 h and scratch wound confluence was calculated. We assessed the effect of siRNA-mediated NOX5 knockdown in combination with v-ATPase inhibition with bafilomycin A and siRNA-mediated knockdown of V1C1 in combination with the pan-NOX inhibitor diphenyleneiodonium (DPI). NOX5 is the only NOX family member expressed in PSN-1 cells as assessed by digital PCR (Fig. 5*B*). As observed previously, siRNA-mediated knockdown of NOX5 decreased PSN-1 cell migration as assessed by the wound confluence timecourse (Fig. 5*C*) and the wound confluence at 18 h (Fig. 5*D*). Pretreatment of PSN-1 cells with 100 nM bafilomycin A for 6 h resulted in a greater decrease in cell migration than with NOX5 knockdown, and knockdown of NOX5 combined with bafilomycin A pretreatment had no additional effect (Fig. 5, *C* and *D*). We have found that commercial antibodies to NOX5 are not always consistent in recognizing endogenous protein. Therefore, the efficacy of NOX5 knockdown was assessed using digital PCR (Fig. 5*E*). V1C1 protein levels were not affected by NOX5 knockdown (Fig. 5*F*). The siRNA-mediated knockdown of V1C1 (Fig. 5, *I* and *J*), and treatment with DPI (1 μM DPI added at the start of the scratch assay timecourse) both resulted in a decrease in PSN-1 cell migration, and together, they had a combined effect (Fig. 5, *G* and *H*). However, digital PCR analysis indicated that both treatments (V1C1 knockdown and DPI treatment) altered NOX5 expression: V1C1 knockdown decreased NOX5 mRNA, DPI treatment increased NOX5 mRNA, and the combined knockdown and inhibition returned NOX5 mRNA levels to baseline (Fig. 5*K*). These unexpected changes in NOX5 expression make interpretation of these experimental conditions difficult.

**Figure 5:**
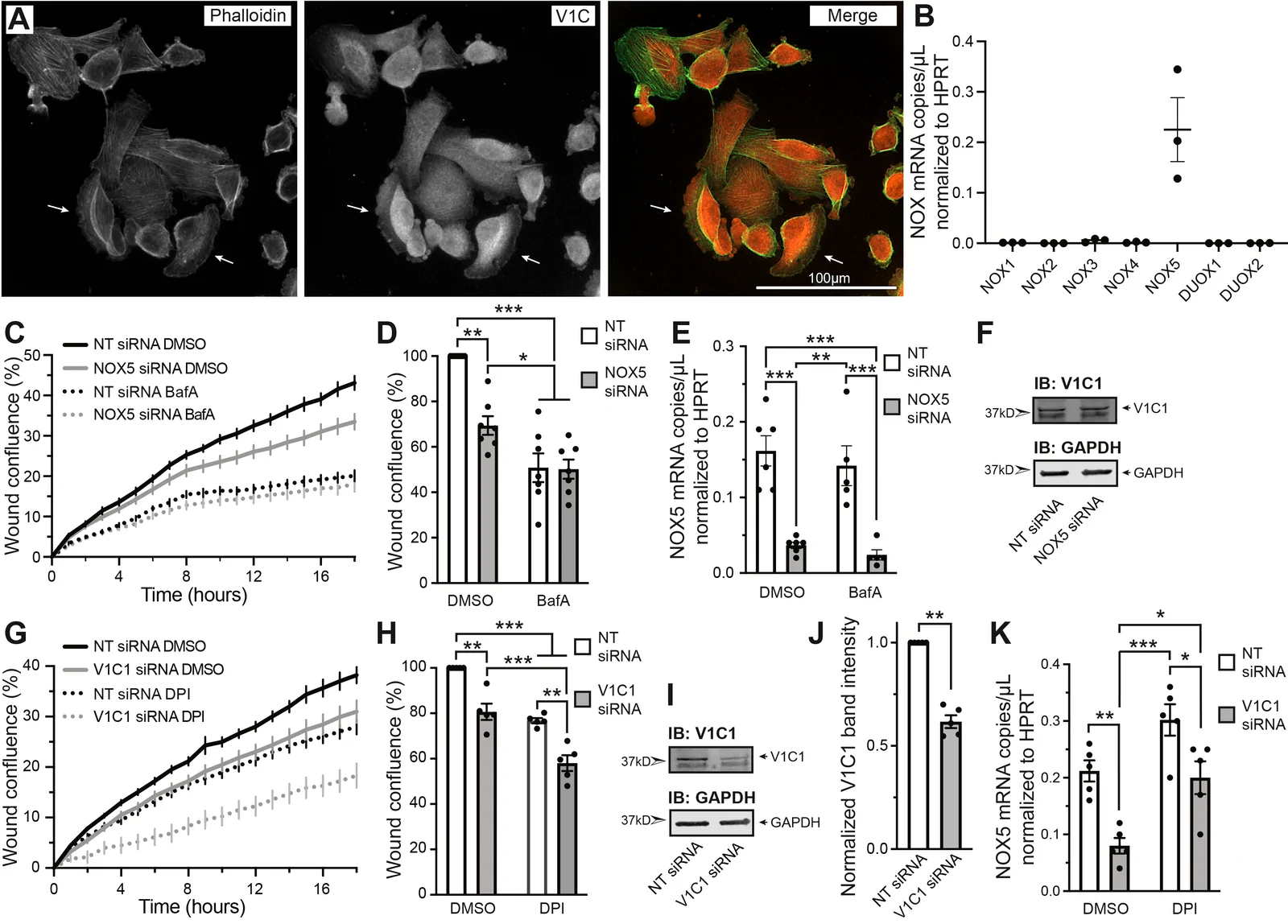
**NOX5 and thev-ATPase contribute to PSN-1 cell migration.***A*, confocal immunofluorescent microscopy of V_1_ subunit V1C1 with F-actin staining with phalloidin in PSN-1 cells. *Grayscale images* are of individual channels, and merged image displays phalloidin in *green* and anti-V1C in *red*. The scale bar represents 100 μm, and *white arrows* indicate regions of the cell leading edge. *B*, digital PCR (dPCR) measurement of NOX family mRNA in PSN-1 cells normalized to housekeeping gene hypoxanthine guanine phosphoribosyltransferase (HPRT). Values represent means ± SEM, n = 3. *C*, timecourse of PSN-1 wound confluence after scratch with quantification of wound confluence at 18 h shown in (*D*). Prior to scratch, cells were transfected with nontargeting (NT) or NOX5-targeting siRNA and treated with DMSO (vehicle control) or bafilomycin A (BafA) for 6 h. Values represent means ± SEM, n = 7, with differences assessed by one-way ANOVA with Tukey’s test, ∗ denoting *p* < 0.05, ∗∗*p* < 0.01, and ∗∗∗*p* < 0.001. *E*, NOX5 mRNA levels assessed by dPCR at conclusion of scratch assay normalized to housekeeping gene HPRT. Values represent means ± SEM, n = 5, with differences assessed by one-way ANOVA with Tukey’s test, ∗∗ denoting *p* < 0.01, and ∗∗∗*p* < 0.001. *F*, representative Western blot of V1C1 and GAPDH with NT and NOX5-targeting siRNA transfection in PSN-1 cells. *G*, timecourse of PSN-1 wound confluence after scratch with quantification of wound confluence at 18 h shown in (*H*). Prior to scratch, cells were transfected with nontargeting (NT) or V1C1-targeting siRNA and treated with DMSO (vehicle control) or diphenyleneiodonium (DPI, added after scratch). Values represent means ± SEM, n = 5, with differences assessed by one-way ANOVA with Tukey’s test, ∗∗ denoting *p* < 0.01, and ∗∗∗*p* < 0.001. *I*, representative Western blot of V1C1 and GAPDH with NT and V1C1-targeting siRNA transfection with quantification in (*J*). Values represent means ± SEM, n = 5, with differences assessed by two-tailed tailed Student’s *t* test with ∗∗ denoting *p* < 0.001. *K*, NOX5 mRNA levels assessed by dPCR at the conclusion of the scratch assay, normalized to the housekeeping gene HPRT. Values represent means ± SEM, n = 5, with differences assessed by one-way ANOVA with Tukey’s test, ∗ denoting *p* < 0.05, ∗∗*p* < 0.01, and ∗∗∗*p* < 0.001. NOX, NADPH oxidase; v-ATPase, vacuolar-type ATPase; V1C1, V1 subunit C1.

## Discussion

Oxidative signaling through NOX-derived ROS is essential for several critical physiological functions; however, dysregulation can lead to oxidative stress, tissue damage, and disease. We recently reported that chemical stabilization of F-actin leads to superoxide production by NOX5 through an unknown mechanism (29). Here, we show that this activation is distinct from previously reported modes of NOX5 activation, including increased intracellular Ca^2+^ levels and NOX5 phosphorylation. Instead, we found that F-actin stabilization led to the formation of a protein complex comprising actin, actin-binding proteins, NOX5, and the v-ATPase, and that chemical inhibition and v-ATPase subunit knockdown reduced extracellular NOX5 superoxide production in response to F-actin stabilization. Finally, NOX5 and the v-ATPase were found to partially localize to the cell leading edge, and combinations of v-ATPase and NOX5 knockdown and inhibition suggest that they coordinate to stimulate cell migration in the pancreatic cancer cell line PSN-1. While previous work has established an essential role for Hν1 in phagocytic NOX ROS production (39, 40) and NOX5 activity in human spermatozoa (41), this work is the first to show a connection between NOX5 and the v-ATPase, that the v-ATPase can stimulate NOX5 superoxide production, and that their previously reported effects on cell migration (29, 31, 52) may be coupled. Moreover, it may represent an additional nexus between oxidative signaling and the cell’s metabolic state.

Figure 6 depicts a model consistent with our results and previously published findings. Increases in F-actin, as at the leading edge of migrating cells, promote the assembly of a protein complex that includes fully assembled v-ATPase and NOX5. F-actin-induced assembly of the v-ATPase leads to protons being pumped across the membrane, and the resulting gradient stimulates electron transfer through NOX5 to produce superoxide. Downstream effects of this localized ROS production, which remain to be fully elucidated, then contribute to directional cell migration. Whether the v-ATPase acts to activate NOX5 superoxide production at other membranes, and if it has a similar stimulating effect on other NOX family members, remains unknown. Moreover, whether the v-ATPase acts as a true activator or instead enhances activation induced by another mechanism remains to be determined. While we hypothesize that the F-actin dependent activation of NOX5 by the v-ATPase is due to the creation of a proton gradient stimulating electron transfer across the PM, several other possibilities exist. Utilizing a v-ATPase inhibitor and v-ATPase subunit knockdown suggest that v-ATPase activity is required for its effect on NOX5, however, we have not completely eliminated the possibility that v-ATPase assembly at the PM in the context of the F-actin associated protein complex is sufficient to support an interaction with an unidentified protein or small molecule activator, or disrupt an inhibitory interaction. Furthermore, whether NOX5 activation is a direct consequence of a v-ATPase generated proton gradient, or due to v-ATPase dependent changes in endocytosis, or other cellular processes, is unknown. These mechanistic details can now be investigated.

**Figure 6:**
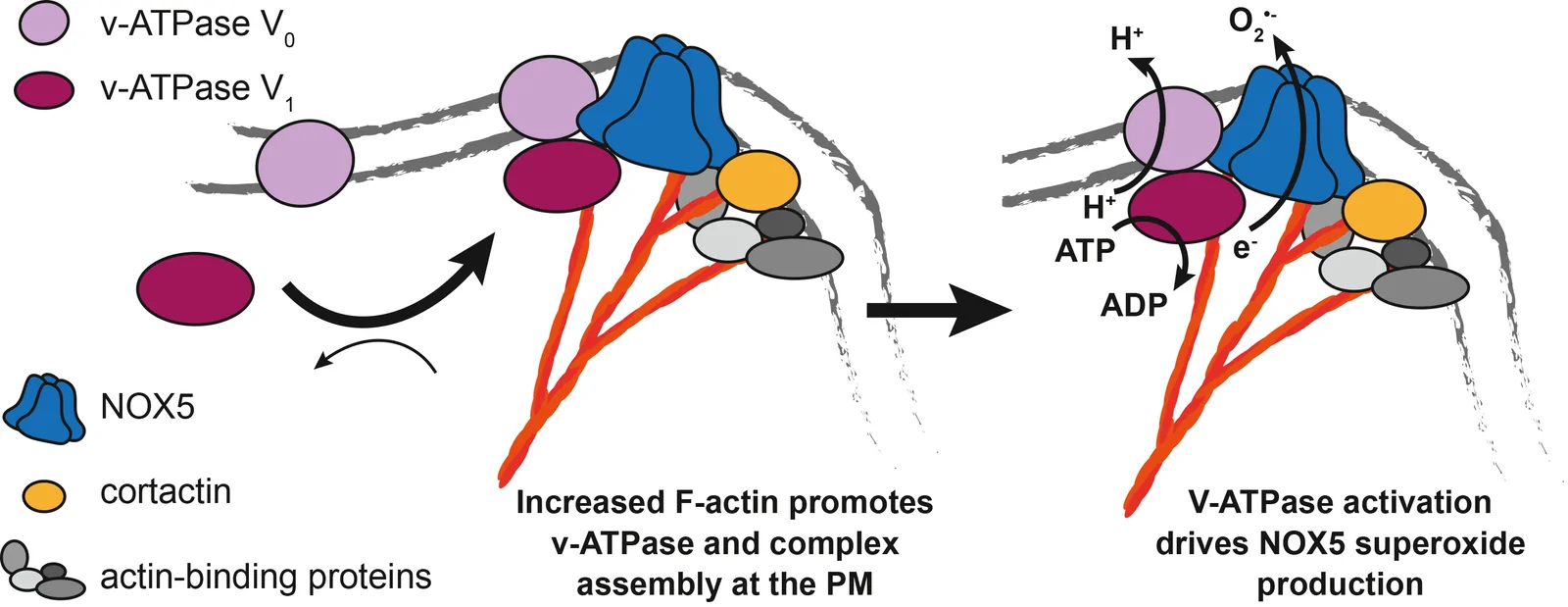
**Proposed model of F-actin-dependent stimulation of NOX5 superoxide production by the v-ATPase.** Increases in F-actin promote v-ATPase assembly at the PM, forming a complex with actin, actin-binding proteins and NOX5. Activity of the v-ATPase then stimulates NOX5 superoxide production. NOX, NADPH oxidase; PM, plasma membrane; v-ATPase, vacuolar-type ATPase.

The v-ATPase is a ubiquitously expressed proton pump that functions in organelle and extracellular acidification. Its best-characterized role is in autophagy, but it also plays critical roles in several other cellular processes, including membrane trafficking, receptor recycling, and coupled ion transport (31, 34, 53). V-ATPase localized to the PM plays a role in bone resorption, sperm maturation, and urine acidification (31, 34, 53), while aberrant activity is associated with cancer invasiveness, neurodegenerative diseases, and diabetes (31, 34, 53). The v-ATPase also plays an important role in nutrient sensing, and its activity has been shown to be regulated by glucose levels (31, 34, 36, 53, 56). Intriguingly, elevated glucose has also been shown to increase NOX5 expression levels (12), suggesting a broad integration between NOX5 oxidative signaling, v-ATPase activity, and nutrient state. Activity of the v-ATPase is considered to be directly coupled to its assembly (31, 32, 33, 34, 35, 36, 37, 38) and NOX5 (29) and the v-ATPase V_1_ subunits B and C (49, 50, 51) have been shown to bind to the actin cytoskeleton. Our data suggest that increases in F-actin bring NOX5, fully assembled v-ATPase, and an array of actin-binding proteins together at the cell membrane, and that this complex contributes to cell migration. Although we have revealed some aspects of the complex, such as an interaction between NOX5 and cortactin, but not between the v-ATPase and cortactin, several details, including stoichiometry, specifics of direct protein–protein interactions, and which isoforms of the v-ATPase are involved, remain unknown. Future work can now investigate the details of this complex and its dynamics. Since aberrant activity of several NOX family members (57) and PM localized v-ATPase (31) have been shown to contribute to cancer invasiveness, the interface(s) driving assembly of this complex and mediating its effects on cell migration could represent an effective target for cancer therapeutics.

Interestingly, we identified a jasplakinolide-induced enrichment of mitochondrial translation machinery in our glycerol gradient fraction of interest. A recent paper has linked v-ATPase activity to mitochondrial translation, showing that partial impairment of the v-ATPase can mitigate the deleterious effects of actinonin, a compound that induces mitochondrial mistranslation (48). Whether there is a direct interaction between the v-ATPase and mitochondrial translation machinery, and whether actin and NOX5 (or other NOX family members) are involved, remains to be seen. However, these questions can now be addressed.

There are known associations between NOX activity and processes related to the v-ATPase, including phagosomal function (58, 59, 60) and autophagy (61), with NOX family members exerting their effects by modulating pH levels (58), and directly oxidizing protein targets (59). However, this is the first work to show that the v-ATPase contributes to NOX5 superoxide production. Notably, this effect is specific to F-actin dependent activation of NOX5, in contrast to activation by increases in intracellular Ca^2+^ levels or NOX5 phosphorylation, as v-ATPase inhibition with bafilomycin A, or knockdown of the V_1_ subunit V1C1 both decreased NOX5 superoxide in response to jasplakinolide treatment, while activation by ionomycin Ca^2+^ was unaffected by bafilomycin A and only slightly decreased by V1C1 knockdown, and activation by PMA was unaffected by V1C1 knockdown, and only slightly decreased by bafilomycin A. This suggests that while the F-actin-dependent stimulation of NOX5 superoxide production is mediated by the v-ATPase, the effects of v-ATPase inhibition and knockdown on NOX5 stimulation by Ca^2+^ and phosphorylation are due to off-target or indirect effects. A further confounding factor is the effect of v-ATPase inhibition and subunit knockdown on NOX5 mRNA and protein levels. We found that after 24 h of bafilomycin A treatment, NOX5 protein levels were decreased. More surprisingly, after 30 h of V1C1 knockdown, NOX5 mRNA levels were also decreased. How these effects on NOX5 expression are mitigated, and whether similar effects occur in cells expressing other NOX family members, remains unknown; however, this may represent an additional layer of v-ATPase regulation of NOX ROS production.

Several NOX family members, as well as the v-ATPase, have been shown to contribute to cell migration (29, 31, 52, 53, 62), however, this is the first work to show that they may be collaborating in this process. We found that both NOX5 knockdown and, to a greater extent, inhibition of the v-ATPase with bafilomycin A, decreased PSN-1 cell migration, but that knockdown of NOX5 in addition to bafilomycin A treatment had no further effect. This suggests that the effect of the v-ATPase on cell migration is upstream of NOX5. Conversely, combining V1C1 knockdown and NOX5 inhibition with DPI appeared to have an additive effect; however, the unexpected effects of V1C1 knockdown on NOX5 mRNA levels complicate the interpretation of this experiment. Several proteins essential for cell migration have been shown to be redox regulated (63). Therefore, we suggest a model in which 1) increases in F-actin at the cell leading edge stimulates assembly of a large protein complex, including the v-ATPase and NOX5, 2) activation of the v-ATPase stimulates NOX5 superoxide production, 3) this superoxide is converted to hydrogen peroxide by extracellular superoxide dismutase and reenters the cell, 4) reversible oxidative modification of redox regulated proteins associated with cell migration occurs, altering their activity on the actin cytoskeleton, and 5) this leads to a combination of localized F-actin disassembly, and direct oxidative modification of the protein complex components (potentially including v-ATPase subunits), thereby resetting the system to allow for the iterative rounds of actin dynamics necessary for cell migration. Future work can now focus on assessing this model and determining the downstream effects of the v-ATPase and NOX5 at the PM to elucidate details of their contributions to cell migration and to identify new therapeutic targets for cancer and other human diseases involving aberrant cell migration associated with the v-ATPase and NOX5.

There has been significant interest in developing NOX family member-specific inhibitors, with some recently entering clinical trials. There is also significant interest in inhibiting disease-associated activities of the v-ATPase. Here, we present our findings demonstrating the interaction between NOX5 and the v-ATPase at the cell leading edge, the role of the v-ATPase in F-actin-dependent activation of NOX5 superoxide production, and their combined effects on cell migration. Several open questions remain, including whether this interaction also occurs in intracellular membranes, whether it is specific to NOX5 or relevant to other NOX family members, the molecular details of the protein complex associated with F-actin at the cell leading edge, and the mechanistic steps linking complex formation, localized activation of the v-ATPase and NOX5, and their effects on cell migration. This work has laid the foundation for answering these questions and identified a novel target for directed therapeutic development.

## Experimental procedures

### Cell culture and transient transfection

HEK293 cells (HEK cells immortalized by transfection with adenovirus 5, American Type Culture Collection [ATCC]) and HEK293 cells stably expressing NOX5β (64) were cultured at 37 °C with 5% CO_2_ in a humidified incubator in Dulbecco’s modified Eagle’s media (DMEM) with 10% fetal bovine serum (FBS) and either antibiotic-antimycotic (Gibco, 1X, HEK293 cells) or G418 (Gibco, 400 μg/ml, HEK293 cells stably expressing NOX5β). PSN-1 cells (human pancreatic cancer cell line derived from adenocarcinoma (65), ATCC) were cultured in RPMI 1640 (Cytiva) with 10% FBS antibiotic-antimycotic (Gibco, 1X) in the same incubation conditions as previously. For maintenance and all uses PSN-1 cells were dissociated using TrypLE (Gibco).

For transfections, HEK293 cells were grown in antibiotic-free DMEM with 10% FBS, then transfected with 5 μg DNA (empty vector, or NOX5β, Addgene) and 15 μl Lipofectamine 2000 (Thermo Fisher Scientific) per 10 cm dish. After 16 h of transfection, cell media was changed to DMEM with 10% FBS and antibiotic-antimycotic. HEK293 cells stably expressing NOX5β were dissociated using TrypLE and plated for transfection in antibiotic-free DMEM with 10% FBS. These cells were transfected using 30 μl of Lipofectamine RNAiMax and 5 μl of 20 μM siRNA per 10 cm dish, with either a scramble nontargeting sequence or V1C1-targeting (Thermo Fisher Scientific), ABL1-targeting (Dharmacon), or PRKCA-targeting (Dharmacon). After 16 h of transfection, cell media was changed to DMEM with 10% FBS and G418. For transfection of PSN-1 cells, cells were grown in antibiotic-free RPMI 1640 media with 10% FBS and transfected with 30 μl of Lipofectamine RNAiMax and 5 μl of 20 μM siRNA per 10 cm dish, with either a scramble nontargeting sequence or NOX5-targeting (Dharmacon) or V1C1-targeting (Dharmacon). After 16 h of transfection, cell media was changed to RPMI 1640 with 10% FBS and antibiotic-antimycotic.

### Superoxide measurements

Cells were transfected and or treated as described and plated in phenol red-free DMEM/F-12 media with 15 mM Hepes and L-glutamine with 10% FBS and 400 μg/ml G418. A total of 2 × 10^5^ cells were plated in each well of a white clear bottom tissue culture-treated 96-well plate (Thermo Fisher Scientific) approximately 2 h prior to assay to achieve a density of 90% or greater at the time of assay. L-012 (Wako Chemicals) was then added to each well at a concentration of 400 μM for 20 min prior to the assay. Assay was performed by measuring luminescence using a Flexstation3 (Molecular Devices). NOX5 superoxide production was measured every 6 s with automated addition of 1 μM ionomycin Ca^2+^ (Sigma-Aldrich) or 1 μM jasplakinolide (R&D Systems). In addition, separate readings were obtained for 40 or 120 min following manual addition of 100 nM PMA (R&D Systems) or 1 μM jasplakinolide, with luminescence values read and recorded every 45 s to observe extended methods of activation. Data were plotted and analyzed using GraphPad Prism v10. Completed datasets were assessed for outliers using a Grubbs test.

### Western blot analysis

Cell lysates were normalized to total protein content as determined by a DC protein assay (Bio-Rad). SDS-PAGE and Western blot analysis were performed using standard procedures with antibodies against NOX5 (Proteintech), β-actin (Sigma-Aldrich, Cell Signaling Technology), cortactin (R&D Systems, Proteintech), v-ATPase subunits V1C1, V1A1, or V_0_ complex, D1 (Proteintech), GAPDH (Proteintech), PKCα (Proteintech), c-Abl (Sigma-Aldrich), or the sodium potassium pump (Cell Signaling Technology). Proteins were visualized using fluorescently labeled secondary antibodies (anti-rabbit and anti-mouse, LI-COR Biosciences). Antibodies were validated with knockdown (endogenous expressing proteins) or empty vector *versus* protein transfection (NOX5 expression in HEK293 cells). Western blots were imaged using a LI-COR Odyssey and analyzed using Empiria Studio 2.2 software (LI-COR Biosciences). For quantified data, completed datasets were assessed for outliers using a Grubbs test.

### Immunoprecipitation assay

HEK293 and HEK293 cells stably expressing NOX5β were cultured as described, then lysed with Cellytic M lysis buffer (Sigma-Aldrich) containing a protease inhibitor (Sigma-Aldrich) or protease/phosphatase inhibitor (Cell Signaling Technology, for phosphoproteomics). Protein concentrations were determined by DC Protein Assay kit (Bio-Rad) per manufacturer’s instructions. A total of 1000 μg of lysate was combined with 3 μg mouse anti-ATP6V1A antibody (Proteintech), 3 μg rabbit anti-ATP6V1C1 antibody (Proteintech), 2 μg goat anti-cortactin antibody (R&D systems), or 3.2 μg rabbit anti-NOX5 antibody (Proteintech, for phosphoproteomics). PBS (Sigma-Aldrich) containing protease inhibitor (Sigma-Aldrich), or protease/phosphatase inhibitor (Cell Signaling Technology, for phosphoproteomics) was added to reach a total volume of 500 μl. Samples were incubated on a rotating mixer at 4 °C for 2 to 3 h. After rotation, samples were added to a tube containing 50 μl of prewashed Protein G Mag Sepharose beads (Cytiva) for mouse antibodies or Protein A Sepharose beads (Thermo Fisher Scientific) for rabbit antibodies. The sample and bead mixtures were incubated on a rotating mixer at 4 °C for 16 h. After incubation, the supernatant was removed using a magnetic rack (Protein G Mag Sepharose beads) or centrifugation for 3 min at 1000 RPM (Protein A Sepharose beads), and the beads were washed 3 times with PBS (Sigma-Aldrich). A total of 30 μl of 2× sample buffer (Bio-Rad) was added to each sample, boiled at 100 °C for 10 min and subjected to SDS-page and Western blot analysis as described previously. For phosphoproteomic samples, on-bead digestion was performed.

### Phosphoproteomics

HEK293 cells stably expressing NOX5β were cultured as described previously and treated for 5 min with 1 μM jasplakinolide, 100 nM PMA, or DMSO (×2). After treatment, cells were lysed and protein lambda phosphatase (New England Biolabs) was added to one of the DMSO-treated samples. Immunoprecipitation with an anti-NOX5 antibody was performed as described previously. The beads were then resuspended in 100 μl of 40% Invitrosol and 100 mM ammonium bicarbonate with 1% Sigma phosphatase inhibitor cocktail 3. Cysteines were reduced with 5 mM Tris(2-carboxyethyl)phosphine hydrochloride for 30 min at 37 °C and alkylated with 10 mM iodoacetamide for 30 min at 37 °C. The bead-bound proteins were then digested overnight with 5 μg of trypsin at 37 °C, followed by cleanup using the SP2 method and drying under vacuum. This material was dissolved in 150 μl of binding/equilibration buffer from the Pierce High-Select TiO2 kit and processed according to the manufacturer’s protocol. The eluted material was dried, and the unbound and wash fractions were combined and dried. The unbound material was redissolved in 200 μl of binding/equilibration buffer from the Pierce High-Select Fe-NTA kit and processed according to the manufacturer’s protocol and the eluted material was dried. Each sample was analyzed on a Thermo Fisher Scientific Orbitrap Fusion Lumos MS *via* two technical replicate injections using a data-dependent acquisition HCD MS2 instrument method. MS data were analyzed using Proteome Discoverer 3.3 (Thermo Fisher Scientific) platform. Raw files were converted to mzML files using ProteoWizard msConvert (66) and uploaded to the MassIVE proteomic repository (MSV000102911).

### Glycerol gradient fractionation and MS

HEK293 cells stably expressing NOX5β were cultured as described previously and treated for 5 min with 1 μM jasplakinolide, 100 nM PMA, or DMSO (x2). After treatment, cells were lysed, and lambda protein phosphatase (New England Biolabs) added to one of the DMSO-treated samples. Protein concentrations were determined by DC Protein Assay kit (Bio-Rad) per manufacturer’s instructions and 2.5 mg of protein was mixed with lysis buffer with protease and phosphatase inhibitors for a total volume of 100 to 200 μl. Afterward, fractionation by glycerol gradient was performed. A total of 10 to 40% (v/v) glycerol gradients were prepared using a Gradient Master (Biocomp). Samples were added to prepared gradients and ultracentrifuged in a swinging bucket ultracentrifuge at 25,000 RPM for 24 h at 4 °C. Samples were then fractionated into 35 fractions with a Piston Gradient Fractionator (BioComp Instruments). Proteins were precipitated with ice-cold acetone, incubated for 1 h at −80 °C and then centrifuged at 20,000*g*, for 15 min at 4 °C. Samples were then air-dried in a fume hood for 30 min. Western blotting was performed on odd-numbered fractions 1 to 35. For this, samples were reconstituted with 50 μl of 1 x Laemmeli buffer with β-mercaptoethanol, followed by SDS-PAGE and Western blotting as previously. MS analysis was performed on fraction 22 of each treatment group. Each dry sample was divided into four tubes. A total of 200 μl of 40% Invitrosol, 20% acetonitrile, and 100 mM ammonium bicarbonate was added to the first tube of each, vortexed, sonicated for 10 cycles of 10 s on/30 s off on ice, centrifuged, transferred to the next tube, and the process repeated to combine the contents of the four tubes of each sample. Cysteines were reduced in 5 mM Tris(2-carboxyethyl)phosphine hydrochloride at 37 °C and 1200 RPM mixing for 30 min, alkylated with 10 mM iodoacetamide at 37 °C and 1200 RPM mixing for 30 min in the dark, digested overnight with 5 μg of sequencing grade trypsin (Promega) at 37 °C and 1200 RPM mixing, and desalted using PreOmix Phoenix spin columns according to the manufacture’s protocol. The Pierce Quantitative Fluorescent Peptide Assay (Thermo Fisher Scientific) was used to determine sample concentrations in order to normalize to 500 ng for each 20 μl LC-MS injection, with Thermo Fisher Scientific Pierce Peptide Retention Time Calibration Mixture added at 4 nM concentration. Equal amounts of each were combined to form a pooled QC sample. Each sample was analyzed on a Thermo Fisher Scientific Orbitrap Fusion Lumos MS *via* 3 technical replicate injections using a data-dependent acquisition HCD MS2 instrument method. The technical replicates were blocked, and each block was randomized. Pooled QCs, which is a mixture of all the samples being analyzed, was analyzed at the start, end, and in between each sample. MS data were analyzed using the Proteome Discoverer 2.4 platform (Thermo Fisher Scientific). Protein identifications were filtered to include only those proteins identified by two or more unique peptides identified. Raw files were converted to mzML files using ProteoWizard msConvert (66) and uploaded to the MassIVE proteomic repository (MSV000102916).

### Analysis and visualization of proteins identified *via* MS

Proteins identified as enriched in the jasplakinolide-treated sample relative to DMSO were analyzed using the STRING database for protein-protein association networks (46), and visualized using Cytoscape (47).

### PM isolation

HEK293 cells stably expressing NOX5β (5 × 10^8^ cells per condition) were cultured as described, then lysed with Cellytic M lysis buffer (Sigma-Aldrich) and homogenized using a syringe and needle. The PM and associated proteins were extracted using the Abcam PM protein extraction kit according to the manufacturer’s instructions. Cytoplasmic and PM fractions were subjected to Western blot analysis as described and probed for NOX5 and the sodium/potassium pump.

### Immunofluorescent imaging

HEK293 cells stably expressing NOX5β or PSN-1 cells were grown on microscope cover glass as described previously, then fixed with 4% paraformaldehyde in PBS for 15 min at RT, then washed three times with PBS before blocking for 15 min at RT using 3% donkey serum and 0.3% Triton X-100 in PBS. Afterward, HEK293 cells stably expressing NOX5β were incubated with anti-V1C1 at a 1:250 dilution (Proteintech), anti-cortactin at 1:500 (R&D Systems), and/or anti-NOX5 antibodies at 1:250 (Proteintech). PSN-1 cells were incubated with an anti-V1C1 antibody at a 1:250 dilution. Primary antibody staining was performed in a humidity chamber overnight at 4 °C. Slides were washed three times with PBS before secondary antibody staining at a 1:1000 dilution for 1 h at RT. Phalloidin staining was performed at this time at a 1:400 dilution (Thermo Fisher Scientific). Secondary antibodies used were donkey anti-goat (Thermo Fisher Scientific), donkey anti-rabbit (Thermo Fisher Scientific), or donkey anti-mouse (Thermo Fisher Scientific). Mounting was performed using mounting medium with DAPI (Vector Laboratories), and samples were dried before sealing with nail polish. Imaging was done using a Cytation C10 (Agilent). Antibodies were validated with knockdown (endogenous expressing proteins) or empty vector *versus* protein transfection (NOX5 expression in HEK293 cells).

### Scratch assay

PSN-1 cells were cultured and transfected with siRNA (nontargeting, NOX5-targeting (Dharmacon), or v-ATPase V1C1-targeting (Thermo Fisher Scientific)) as described previously. Cells were then plated into clear 96-well plastic plates (GenClone) 6 h prior to assay to achieve 100% density at the time of launching the assay. For the relevant samples, 100 nM bafilomycin A or DMSO treatment was added at time of plating. The scratch was made using a woundmaker (Essen Bioscience, 96-well plate). Immediately after scratching, media was changed to fresh RPMI 1640 (Cytiva) with 10% FBS and antibiotic-antimycotic (Gibco, 1 X). For the relevant samples, 1 μM DPI (Sigma-Aldrich) or DMSO were added with the fresh media. Data were collected using Agilent Cytation C10 for 18 to 22 h at 37 °C and 5% CO_2_ and analyzed with Gen5 3.12 software. For quantification, each biological replicate was normalized to the matched double control (NT siRNA, DMSO). Completed datasets were assessed for outliers using a Grubbs test.

### Digital PCR

After completion of scratch assays, PSN-1 cells were lysed in RLT buffer (Qiagen) with β-mercaptoethanol (Bio-Rad) and homogenized using QIAshredder columns (Qiagen). Lysates were then processed to extract mRNA (RNeasy kit, Qiagen) and converted to cDNA (high-capacity cDNA reverse transcription kit, Thermo Fisher Scientific). Equal concentrations of cDNA from PSN-1 cells of each treatment group were analyzed using a QIAcuity digital PCR system using the QIAcuity probe kit and the Taqman primer/probe assay kit for NOX family members (Thermo Fisher Scientific). Copies/μl of NOX family transcripts were normalized to hypoxanthine-guanine phosphoribosyltransferase housekeeper expression in copies/μl using the QIAcuity software suite (Qiagen).

### Key resources table

See Table 1.

**Table 1:** Key reagents and resources

Reagent or resource	Source	Identifier
Antibodies		
Rabbit polyclonal anti-NOX5	Proteintech	Cat#25350-1-AP, RRID:AB_2811208
Mouse monoclonal anti-β-actin (AC-15)	Sigma-Aldrich	Cat# A5441, RRID:AB_476744
Rabbit polyclonal anti-β-Actin (13E5)	Cell Signaling Technology	Cat# 4970, RRID:AB_2223172
Goat polyclonal anti-cortactin	R and D Systems	Cat# AF6096, RRID:AB_2044650
Rabbit recombinant monoclonal anti-cortactin	Proteintech	Cat# 84569-1-RR, RRID:AB_3672073
Rabbit polyclonal anti-ATP6V1C1	Proteintech	Cat# 16054-1-AP, RRID:AB_2062501
Mouse monoclonal anti-ATP6V1A	Proteintech	Cat# 68440-1, RRID:AB_3085153
Rabbit polyclonal anti-ATP6V1A	Proteintech	Cat# 17115-1-AP, RRID:AB_2290195
Rabbit polyclonal anti-ATP6V1C1	Proteintech	Cat# 16054-1-AP, RRID:AB_2062501
Mouse monoclonal anti-GAPDH (1E6D9)	Proteintech	Cat# 60004-1, RRID:AB_2107436
Mouse monoclonal anti-PKC alpha	Proteintech	Cat# 66421-1, RRID:AB_2881793
Mouse monoclonal anti-c-Abl	Sigma-Aldrich	Cat# A5844, RRID:AB_258262
Rabbit polyclonal anti-Na,K-ATPase	Cell Signaling Technology	Cat# 3010, RRID: AB_2060983
IRDye 800CW donkey anti-mouse IgG	LI-COR Biosciences	Cat# 926-32212, RRID:AB_621847
IRDye 800CW goat anti-rabbit IgG	LI-COR Biosciences	Cat# 926-32211, RRID:AB 621843
IRDye 680RD goat anti-rabbit IgG	LI-COR Biosciences	Cat# 926-68071, RRID:AB_10956166
IRDye 680RD donkey anti-mouse IgG	LI-COR Biosciences	Cat# 926-68072, RRID:AB_10953628
Donkey anti-rabbit IgG (H + L) highly cross adsorbed secondary antibody, Alexa Fluor 594	Thermo Fisher Scientific	Cat# A-21207, RRID:AB_141637
Donkey anti-mouse IgG (H + L) highly cross adsorbed secondary antibody, Alexa Fluor 594	Thermo Fisher Scientific	Cat# A-21203, RRID:AB_2535789
Donkey anti-goat IgG (H + L) cross-adsorbed secondary antibody, Alexa Fluor 488	Thermo Fisher Scientific	Cat# A-11055, RRID:AB_2534102
Chemicals, peptides, and recombinant proteins		
L-012	Wako Chemicals	Cat#120-04891, CAS:143556-24-5
Cellytic M, cell lysis reagent	Sigma-Aldrich	Cat# C2978
EDTA-free protease inhibitor cocktail mini tablet	Sigma-Aldrich	Cat# 11836170001
Antiobiotic-antimycotic	Gibco	Cat#15240062, CAS:3810-74-0, 69-57-8
Geneticin (G418 sulfate)	Gibco	Cat#10131035, CAS:108321-42-2
Ionomycin calcium	Sigma-Aldrich	Cat#I0634, CAS:56092-82-1
Jasplakinolide	R and D Systems	Cat# 2792, CAS:102396-24-7
Bafilomycin A	Cayman Chemical Company	Cat# 11038 CAS:88899-55-2
Protein A-Sepharose 4B	Thermo Fisher Scientific	Cat# 101041
Protein G Mag Sepharose	Cytiva	Cat# 28-9940-08
Taqman gene expression assay (NOX1)	Thermo Fisher Scientific	Cat# 4331182, Assay ID: Hs01071088_m1
Taqman gene expression assay (NOX2)	Thermo Fisher Scientific	Cat# 4331183, Assay ID: Hs00166163_m1
Taqman gene expression assay (NOX3)	Thermo Fisher Scientific	Cat# 4331184, Assay ID: Hs01098883_m1
Taqman gene expression assay (NOX4)	Thermo Fisher Scientific	Cat# 4331185, Assay ID: Hs01379108_m1
Taqman gene expression assay (NOX5)	Thermo Fisher Scientific	Cat# 4331182, Assay ID: Hs00225846_m1
Taqman gene expression assay (DUOX1)	Thermo Fisher Scientific	Cat# 4331182, Assay ID: Hs00213694_m1
Taqman gene expression assay (DUOX2)	Thermo Fisher Scientific	Cat# 4331182, Assay ID: Hs00204187_m1
Phorbol 12-myristate 13-acetate (PMA)	R and D Systems	Cat# 1201 CAS:16561-29-8
Diphenyleneiodonium chloride (DPI)	Sigma-Aldrich	Cat# D2926 CAS:4673-26-1
2-mercaptoethanol	Bio-Rad	Cat# 1610710 CAS:60-24-2
Protease/phosphatase inhibitor cocktail	Cell Signaling Technology	Cat# 5872S
Lambda protein phosphatase	New England Biolabs	Cat# P0753
Critical commercial assays		
DC protein assay kit II	Bio-Rad	Cat# 5000112
RNeasy plus micro kit	Qiagen	Cat# 740
High-capacity cDNA reverse transcription kit	Thermo Fisher Scientific	Cat# 4368814
QIAcuity probe kit	Qiagen	Cat# 250102
Plasma membrane protein extraction kit	Abcam	Cat# ab65400
Deposited data		
Mass spectrometry phosphoproteomics	massIVE	MSV000102911
Mass spectrometry fraction 22	massIVE	
Experimental models: cell lines		
HEK293	ATCC	ATCC#CRL-1573, RRID:CVCL_0045
HEK293, hNOX5, and clone B2	Banfi *et al.*, 2001	N/A
PSN-1	ATCC	ATCC# CRL-3211, RRID: CVCL_1644
Oligonucleotides		
ON-TARGETplus nontargeting pool	Dharmacon	Cat# D-001810-10-20
ON-TARGETplus human NOX5 SMARTpool	Dharmacon	Cat# L-010195-00-0020
ON-TARGETplus human ABL1 siRNA SMARTpool	Dharmacon	Cat# L-003100-00-0010
ON-TARGETplus human PRKCA siRNA	Dharmacon	Cat# L-003523-00-0005
ON-TARGETplus human ATP6V1A (523) siRNA, SMARTpool	Dharmacon	Cat# L-017590-01-005
Silencer validated siRNA V1C1	Thermo Fisher Scientific	Cat# AM51333
Recombinant DNA		
pcDNA3.1-hNOX5	Banfi *et al.*, 2001	Addgene: 69354
Software		
Empiria Studio 2.2	LI-COR Biosciences	v2.3.0.154, RRID: SCR_022512
Prism	GraphPad	v10, RRID:SCR_002798
SoftMax Pro Data Acquisition and Analysis Software	Molecular Devices	v7.1.2, RRID: SCR_014240
Gen5 3.12	Agilent	v3.12, RRID: SCR_017317
QIAcuity software suite	Qiagen	v2.1.7.182
Other		
DMEM, high glucose, pyruvate	Sigma-Aldrich	Cat#D7777
RPMI 1640 Media with L-glutamine	Cytiva	Cat# SH30027.FS
DMEM/F-12 15 mM Hepes, L-glutamine w/o phenol red	Sigma-Aldrich	Cat# D2906
Lipofectamine 2000	Thermo Fisher Scientific	Cat# 11668019
Lipofectamine RNAiMax	Thermo Fisher Scientific	Cat# 13778150
TrypLE	Gibco	Cat# 12605010

## Data availability

Datasets are available on request: The raw data supporting the conclusions of this article will be made available by the authors, without undue reservation.

## Supporting information

This article contains supporting information.

## Conflict of interest

The authors declare that they have no conflicts of interest with the contents of this article.
