# Supplementary material for: F-actin-dependent activation of NADPH oxidase 5 by the vacuolar-type ATPase
Source: J Biol Chem. 2026 Sep 11;302(11):113546. doi: 10.1016/j.jbc.2026.113546 (PMC13677954; doi:10.1016/j.jbc.2026.113546)
Supplement: Supplementary file 1 [file mmc2.docx]

**Figure S1. NOX5 expression and superoxide production.** Superoxide production measured using the luminol analog L-012 in HEK293 cells transiently transfected with empty vector (EV) or NOXβ after A) automated addition of DMSO (vehicle control), 1 μM ionomycin Ca^2+^ or 1 μM jasplakinolide (measured for 300 seconds) or B) manual addition of DMSO, 100 nM PMA or 1 μM jasplakinolide (measured for 40 minutes). C) Representative western blot of NOX5 and β-actin in HEK293 cells, HEK293 cells stably expressing NOXβ, and HEK293 cells transiently transfected with EV or NOXβ. D) Representative western blot of calcium/calmodulin-dependent protein kinase type II subunit alpha (CAMKIIα) and GAPDH in HEK293 cells, HEK293 cells stably expressing NOXβ, and PSN-1 cells.

**Figure S2. NOX5 superoxide production after 24 hour bafilomycin A treatment.** Timecourse of superoxide production by HEK293 cells stably expressing NOX5β with automated addition of A) 1 μM ionomycin calcium or B) 1 μM jasplakinolide (300s), or manual addition of D) 100 nM PMA or E) 1 μM jasplakinolide (120 minutes), after pretreatment with DMSO (vehicle control) or 100 nM bafilomycin A (BafA) for 24 hours, measured by L-012 luminescence. (C,F) Quantification of NOX5 superoxide production (area under the curve). Values represent means ± SEM, n=5. Differences in superoxide after BafA treatment compared to DMSO were assessed using a two-tailed student’s t-test with * denoting p < 0.05, ** p < 0.001 and *** p < 0.0001. G) Representative western blot probed for NOX5 and β-actin levels after treatment with DMSO or bafilomycin A for 24 hours.

**Figure S3. Intracellular calcium.** Changes in intracellular calcium measured using the FLIPR Calcium 6-QF assay after automated addition of 1 μM ionomycin calcium (A) or 1 μM jasplakinolide (B) to HEK293 cells stably expressing NOX5β after transfection with non-targeting (NT) or ATP6V1C1-targeting siRNA.

**Table S1. Proteins in glycercol gradient fraction 22 identified by mass spectrometry.** This table is provided as a separate excel file titled “JBC_NOX5_VATPase_TableS1”. Tab 1: Jas(3) vs DMSO(2), lists proteins enriched in jasplakinolide treated sample compared to DMSO treated sample. Tab 2: All proteins, lists all proteins found in fraction 22 for both samples.
